# Effect of Reduction Sequence during Rolling on Deformed Texture and Anisotropy of Ferritic Stainless Steel

**DOI:** 10.3390/ma16103767

**Published:** 2023-05-16

**Authors:** Sang Heon Cho, Young Jin Lee, Warda Bahanan, Jeong Moo Oh, Dong-Ju Kim, Jee-Hyun Kang, Jungho Ryu, I Putu Widiantara, Young Gun Ko

**Affiliations:** 1 School of Materials Science and Engineering, Yeungnam University, Gyeongsan 38541, Republic of Korea; 2 Hyundai BNG Steel Co., Ltd., Changwon 51707, Republic of Korea; 3 SeA Mechanics Co., Ltd., Gumi 39379, Republic of Korea

**Keywords:** stainless steel, rolling, texture, anisotropy, reduction sequence

## Abstract

This investigation studied the effect of reduction sequence during rolling of ferritic stainless steel on texture and anisotropy. A series of thermomechanical processes were performed on the present samples utilizing rolling deformation, with a total height reduction of 83% but with different reduction sequences, 67% + 50% (route A) and 50% + 67% (route B). Microstructural analysis showed that no significant difference was found in terms of the grain morphology between route A and route B. In terms of the texture, as compared to route A, route B developed a sharper texture on all components along the γ-fiber and a considerably higher fraction of boundaries that displayed $38{}^{\circ}\langle 111\rangle$ misorientations with respect to the surrounding deformed grains. In consequence, optimal deep drawing properties were achieved, where *r*_m_ was maximized and Δ*r* was minimized. Moreover, despite the similar morphology between the two processes, the resistance toward ridging was improved in the case of route B. This was explained in relation to the selective growth-controlled recrystallization, which favors the formation of microstructure with homogeneous distribution of the <111>//ND orientation.

## 1. Introduction

For structural purposes, stainless steel (STS) has been used as one of the most sustainable pillar metals in a number of applications, such as mobility, architecture, and electronic devices, due to its decent thermal conductivity, low thermal expansion, and fair corrosion resistance, even under harmful environments [1,2]. The most useful forms have been classified into austenite- and ferrite-based STS with distinctive chemical compositions. Despite the fact that austenitic STS has long held the position, commercial attention is currently being paid to the development of ferritic STS that does not possess expensive transition metals, such as Ni, so as to drive down the cost [3,4]. For manufacturing processes, ferritic STS was fabricated in the form of a sheet (or plate) deformed by cold-rolling followed by annealing for strain relaxation [5,6].

During Sendzimir rolling of ferritic STS sheets at ambient temperature, the appreciable change in deformed structure, such as grain refinement, interface softening, and macro- and micro-texture evolution, was pronounced [7], leading to the variation of mechanical characteristics, such as strength, ductility, and plastic anisotropy that was closely related to drawability. Here, excellent drawability would be achieved by controlling the formation of α-, γ-, ε-fibers, etc. by means of thermomechanical treatment where strain and thermal input are conjugated. In general, the formability characteristics of ferritic STS would be improved by *r*-value (Lankford parameter), which is affected by how both grain flow and preferred orientation are intensified. It was reported that the occurrence of *γ*-fibers during suitable thermal treatment between rolling passage would give rise to an increase in the *r*-value [8]. Ray et al. [9] reported that the cold-formability would be improved with an increasing *r*-value associated with γ-fiber ({111}//ND) as recrystallization texture. Shin et al. [10] demonstrated that one of the γ-fiber components, {111}<$1\bar{1}0$>, weakened the anisotropy and affected the ridging due to its interaction with its neighboring grain.

Up to now, a number of investigations have been conducted by taking a variety of processing variables found in rolling and annealing into account [11,12,13]. Nevertheless, the effect of rolling history (so-called reduction sequence), during rolling under the condition that the total height reduction remains constant, on the relation between texture and anisotropy of ferritic STS has rarely been investigated. Therefore, two different rolling routes (67% + 50% vs. 50% + 67%, total height reduction = 83%) are applied to ferritic STS in this study, and both results are compared in relation to orientation distribution function (ODF) and plastic anisotropy at room temperature.

## 2. Materials and Methods

The chemical composition of the ferritic STS studied in the present research is shown in Table 1.

Two different kinds of routes and their processing history, which includes 1st rolling, intermediate annealing (IA), 2nd rolling, and final annealing (FA), are shown in Table 2. Each rolling process was carried out in a number of passes, depending on the amount of height reduction.

**Figure 1:**
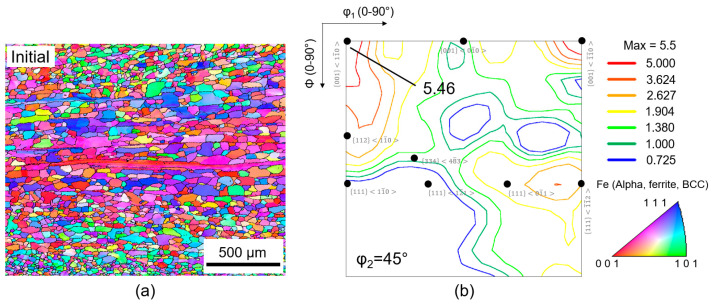
(**a**) Inverse pole figure and (**b**) reduced ODF map of the initial sample of ferritic STS in the central region.

The microstructure and texture of the ferritic STS after processing using route A and route B were examined using electron backscatter diffraction (EBSD) equipped in a scanning electron microscope (SEM). Sheets were cut from the ND-RD (normal direction–rolling direction) plane. A scanning electron microscope (SEM, LEO 1450VP) at 15 kV with a scanning step of ~1.0 μm was used. The results were compared with a database of ferritic phase and further analyzed via the TSL-OIM program. For the initial material, a sheet with a thickness of 3.0 mm was obtained via hot rolling at 925 °C followed by annealing at 950 °C. The microstructure at the central region of the ferritic STS comprises pancake-shaped and equiaxed grains, as shown in Figure 1a. For the texture, the ODF image shown in Figure 1b displayed a pronounced texture consisting of the orientation with an intensity of ~5.46 at $\left\{001\right\}\langle \bar{1}\bar{1}0\rangle$ along the {*hkl*}<110> fiber (or α-fiber). The pancake-shaped grains displayed a pronounced texture consisting of the {001}<*uvw*> or cube orientation, while the equiaxed grains displayed the {110}<*uvw*> or Goss orientation. The samples with a gauge length and width of 50 mm and 20 mm, respectively, were cut-off at angles of 0°, 45°, and 90° from RD. The tension procedures were carried out in room temperature conditions using a Zwick (Z050-50KN) with a strain rate of 0.05 s^−1^. For each result, the experiments were repeated at least five times in order to ensure reproducibility. The anisotropy tests were carried out by straining the sample by 15%, according to the standard of ASTM E517-10. Tensile test routines were performed to measure the *r*_m_ (average normal anisotropy coefficient) and the Δ*r* (planar coefficient of anisotropy) in processed samples. The surface roughness was measured in the transverse direction (TD) of tensile specimens pre-strained by 15% in the RD to evaluate the ridging severity. The surface roughness profile for evaluating ridging was measured at five different positions in the gauge length region of the tensile specimens.

## 3. Results and Discussion

### 3.1. Microstructure Analysis

**Figure 2:**
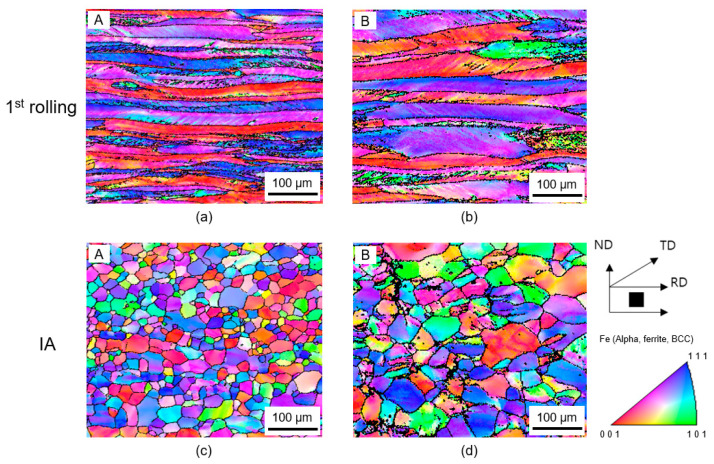
EBSD-IPF images of RD-ND cross sections of the sample after the first rolling for (**a**) route A and (**b**) route B and after IA for (**c**) route A and (**d**) route B.

Figure 2 shows the inverse pole figure (IPF) maps, showing the microstructure of the ferritic STS after the first rolling (Figure 2a,b) and intermediate annealing (Figure 2c,d) via route A and route B. After the first rolling, the microstructure transformed into grains with elongated features along the direction of the rolling deformation with wavy characteristics irrespective of the rolling conditions used in this study, which results in a lamellar-like structure. The average band thickness of the rolled sample after route A (67% height reduction for 5-pass) and route B (50% height reduction for 3-pass) was 27 and 38 μm, respectively (Figure 2a,b). Both microstructures dominated by deformation-textured grains consisted of {100}//ND (red-colored grains) and {111}//ND (blue-colored grains), which was typical of the microstructure of STS after rolling deformation [14]. The fraction of {100}//ND-oriented grains in route A was slightly higher as compared to route B. This was in accordance with a previous study showing that the increase in rolling reduction lead to the increased densities of {100}//ND-oriented grains [15].

After IA at 950 °C for 45 s, most of the grains exhibited a reasonably equiaxed shape (Figure 2c,d). The average grain size was smaller in the sample processed via route A (≈31 μm) as compared to that processed via route B (≈57 μm), which was in accordance with the smaller band thickness in route A as compared to route B.

**Figure 3:**
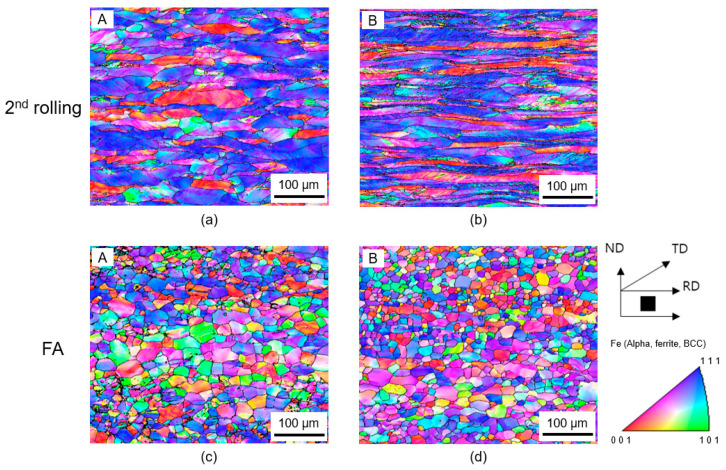
IPF maps of RD-ND cross sections of the sample after the second rolling for (**a**) route A and (**b**) route B and after FA for (**c**) route A and (**d**) route B.

After the second rolling (Figure 3a,b), route B (67% height reduction for 5-pass) exhibited an average band thickness of ~15 μm, which was smaller to route A (50% height reduction for 3-pass), which showed an average band thickness of ~27 μm. In contrast to the first rolling, whose texture was dominated by {100}//ND, the second rolling was dominated by {111}//ND for both routes. Despite the difference in thickness reduction, the fraction of grains with {111}//ND orientation between the two samples was quite similar, as suggested by Keichel et al. [16]. After FA (Figure 3c,d), most of the grain morphology had become equiaxed. The microstructure revealed that the average grain size of route B (≈40 μm) was slightly finer than that of route A (≈48 μm). It can be seen that the fraction of grain with {111}//ND orientation in route B was higher than that in route A. Similarly to the results reported by Engler et al. [17], the fraction of grain with {111}//ND orientation is in accordance with the amount of height reduction such that the fraction of {111}//ND orientation in route B with a higher rolling reduction was larger than that of route A with a lower rolling reduction. The fraction of {001}//ND was significantly decreased in both cases.

### 3.2. Texture Analysis

**Figure 4:**
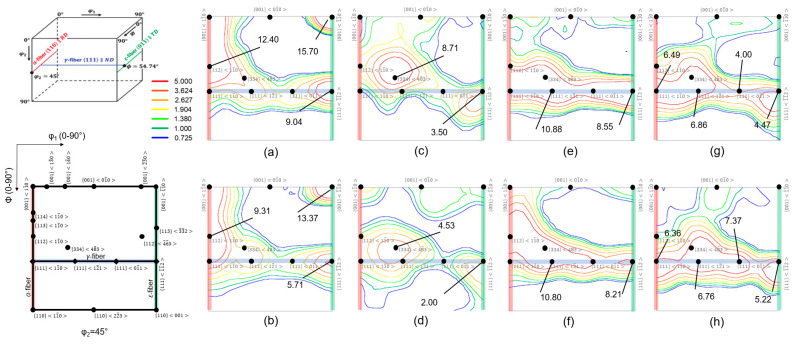
Reduced ODF sections ($\varphi_{2}=45{}^{\circ}$) of the sample after the first rolling for (**a**) route A and (**b**) route B, IA for (**c**) route A and (**d**) route B, second rolling for (**e**) route A and (**f**) route B, and FA for (**g**) route A and (**h**) route B.

ODF maps of ferritic STS with a BCC structure revealed the texture evolution of the microstructure from the first rolling to FA following route A and route B (Figure 4). In this study, the reduced Euler space with $\varphi_{1}:0-90{}^{\circ},\;\Phi =0-90{}^{\circ},\;\varphi_{2}=45{}^{\circ}$ was selected. Several texture components were shown along a number of fibers, namely, alpha fiber (along *ϕ* on $\varphi_{1}=0{}^{\circ}$ and $\varphi_{2}=45{}^{\circ}$), gamma fiber (along $\varphi_{1}$ on ϕ=55° and $\varphi_{2}=45{}^{\circ}$), and epsilon fiber (along *ϕ* on $\varphi_{1}=90{}^{\circ}$ and $\varphi_{2}=45{}^{\circ}$).

After the first rolling (Figure 4a,b), the intensity was strong on the α- and γ-fiber orientations, with a maximum intensity at the rotated cube or $\left\{001\right\}\langle 1\bar{1}0\rangle$, which is typical in ferritic steel after rolling and annealing [17,18]. High densities of orientations were detected in a continuous manner along the α-fiber with a maximum between $\left\{001\right\}\langle 1\bar{1}0\rangle$ and $\left\{111\right\}\langle 1\bar{1}0\rangle$. Based on the current observation, the change in densities of both $\left\{001\right\}\langle 1\bar{1}0\rangle$ and $\left\{111\right\}\langle \bar{1}\bar{1}2\rangle$ seemed to increase with increasing height reduction. After IA (Figure 4c,d), the intensity shifted from their position, especially in the case of the *γ*-fiber. In both processes, a peak on $\left\{334\right\}\langle 4\bar{8}3\rangle$ was developed. This might be associated with the existence of some $\left\{112\right\}\langle 1\bar{1}0\rangle$ peaks, which triggered the formation of $\left\{334\right\}\langle 4\bar{8}3\rangle$ through the phenomenon of selective particle drag [19]. After the second rolling (Figure 4e,f), the strong rotated cube of the ordinarily rolled sheet was successfully removed. The change in the densities of $\left\{111\right\}\langle \bar{1}\bar{1}2\rangle$ was higher in route B as compared to route A, which was expected since route B now has a higher rolling reduction of 67% as compared to that of 50% in route A. For both routes, most orientations were along the *γ*-fiber. Similar observations have been reported previously in the case of a rolled ferritic STS [19,20]. After FA (Figure 4g,h), route B with a higher rolling reduction led to a homogeneous rolling texture, where all components along the *γ*-fiber appeared. In route B, the $\left\{111\right\}\langle 1\bar{1}0\rangle$, $\left\{111\right\}\langle 1\bar{2}1\rangle$, $\left\{111\right\}\langle 0\bar{1}1\rangle$, and $\left\{111\right\}\langle \bar{1}\bar{1}2\rangle$ components have an intensity higher than five. However, in route A, the intensity of $\left\{111\right\}\langle 0\bar{1}1\rangle$ was low.

**Figure 5:**
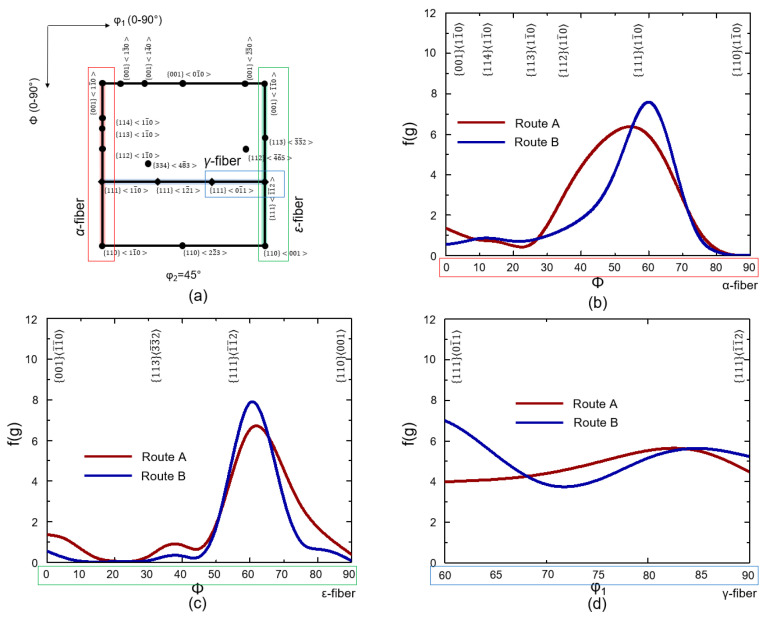
ODF intensity distributions carried out on reduced section ($\varphi_{2}=45{}^{\circ}$) with (**a**) details of analysis range on α-fiber (red outline), ε-fiber (green outline), and γ-fiber (blue outline). ODF intensity distribution for analysis range on (**b**) α-fiber, (**c**) ε-fiber, and (**d**) γ-fiber in route A and route B.

The final texture was formed after all the processes were depicted in detail from the intensity distribution along each fiber, namely, α-, ε-, and γ-fibers (Figure 5a). For the *α*-fiber, as shown in Figure 5b, the distribution of orientation intensity was more localized in the case of route B, so its peak was higher as compared to that of route A. Observation along the ε-fiber (Figure 5c) showed that the intensity was actually shifted from the ideal position of $\left\{111\right\}\langle \bar{1}\bar{1}2\rangle$ and tended to move downward along the *ϕ*-axis, which is in line with a previous report [21]. Here, a similar tendency occurred where the peak density of orientation in route B was higher than that of route A. Along the γ-fiber (Figure 5d), in the final annealed samples, the $\left\{111\right\}\langle 0\bar{1}1\rangle$ densities are 1.8 times higher in route B than in route A, which is in line with the previous ODF analysis.

### 3.3. CSL Boundary Analysis

**Figure 6:**
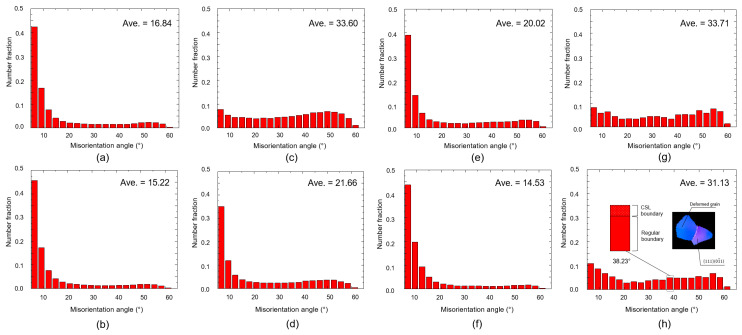
Misorientation distribution during the first rolling of (**a**) route A and (**b**) route B, during intermediate annealing of (**c**) route A and (**d**) route B, during second rolling of (**e**) route A and (**f**) route B, and during final annealing of (**g**) route A and (**h**) route B.

Thermomechanical treatment results in the evolution of boundaries in the microstructure, which can be clearly shown by deriving the information from the EBSD data. Figure 6 shows the misorientation distribution of the sample after the first rolling, intermediate annealing, second rolling, and final annealing for both route A and route B.

After the first rolling (Figure 6a,b), the average misorientations between route A and B were slightly different and characterized by the existence of a high fraction of boundaries with a low misorientation angle (~6°) or low angle grain boundaries (LAGBs). The average misorientation for route A was larger than that for route B, which is most likely due to the higher thickness reduction during the first rolling in route A than that in route B. After the first annealing (Figure 6c,d), the difference became more obvious, where the average misorientation of route A (33.60) was higher than that of route B (21.66). Here, a high fraction of LAGBs transformed into high angle grain boundaries (HAGBs) with misorientations higher than 6°. For route B, only a small amount of LAGBs were transformed, which is most probably due to the lack of driving force originating from the low thickness reduction during the first rolling [22]. After the second rolling (Figure 6e,f), the fraction of LAGBs increased again in both cases. The average misorientation in route B remained lower than that of route A. This might be attributed to the fraction of LAGBs formed previously during the first rolling. After final annealing (Figure 6g,h), the average misorientations between A and B were very close to each other. Several interesting points could be drawn from Figure 6g,h. Firstly, although the average grain size in route B was finer than that in route A (Figure 3c,d), the average misorientation in route B was surprisingly lower than that in route A. Secondly, some fractions of the boundary in route B (in this case, the boundary with a misorientation of ~38.23°, as shown in Figure 6h) were characterized by the coincidence site lattice (CSL) boundary. The first point might be associated with a considerable amount of boundary that remains as an LAGB in route B, and the others might be due to the formation of CSL boundaries of Σ7 with a misorientation of ~38.23°, which is close to the average value of misorientation. Interestingly, such boundaries border the deformed grain, with the grain (or nuclei) exhibiting the $\left\{111\right\}\langle 0\bar{1}1\rangle$ orientation.

**Figure 7:**
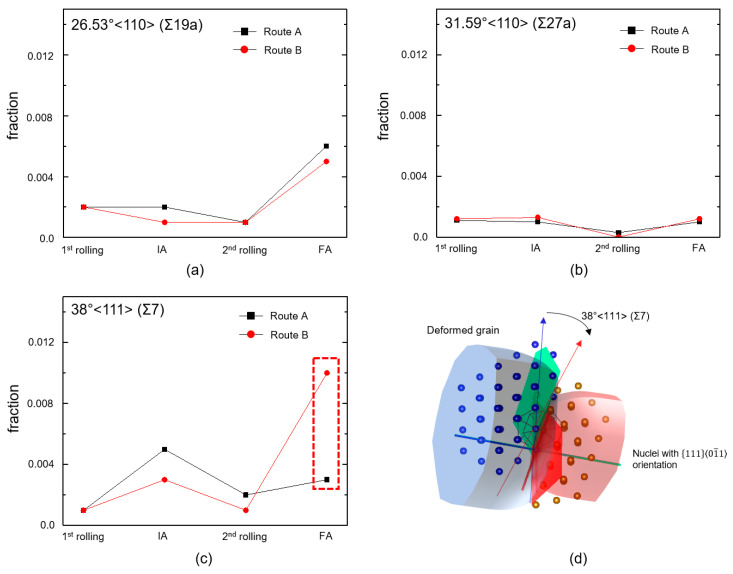
Fraction of CSL boundaries of (**a**) $26.53{}^{\circ}\langle 110\rangle$ (Σ19a), (**b**) $31.59{}^{\circ}\langle 110\rangle$ (Σ27a), and (**c**) $38{}^{\circ}\langle 111\rangle$ (Σ7) samples after the first rolling, IA, second rolling, and FA for route A and route B. (**d**) Schematic of Σ7 with potential to trigger selective growth-controlled recrystallization.

The particular nuclei exhibiting CSL boundaries will not inherit their orientation from the deformed neighboring grains (termed as orientation selectivity) [23], which would be beneficial to add more variety in terms of orientation other than the orientation of the deformed grains. In addition, CSL boundaries with rotation axes parallel to especially the $\langle 110\rangle$ axis and $\langle 111\rangle$ axis were favorable for the growth of nuclei [24,25]. Moreover, in the case of Σ7, the growth was expected to be rapid and contributed significantly to final recrystallization textures via the selective growth mechanism [26]. Here, in addition to the observed CSL boundaries of Σ7 (38°〈111〉), two other CSL boundaries, namely Σ19a (26.53°〈110〉) and Σ27a (31.59°〈110〉), which are commonly found during annealing treatment, will be investigated throughout the thermomechanical processes [26]. As shown in Figure 7a,b, no significant difference was found in terms of the fraction of the Σ19a and Σ27a boundaries between route A and route B, respectively. On the other hand, Figure 7c shows that the fraction of Σ7 boundaries in route B was much higher as compared to route A during the final annealing. Manual indexing of the IPF maps revealed that most of the Σ7 boundaries bordered the deformed grain and the nuclei with $\left\{111\right\}\langle 0\bar{1}1\rangle$ orientation, as shown schematically in Figure 7d. This explained the higher densities of $\left\{111\right\}\langle 0\bar{1}1\rangle$ orientation in route B as compared to route A. It was reported previously that Σ7 boundaries might have occurred during recrystallization in the case of microstructure with concentrated deformation texture [26], which was in agreement with our result in Figure 5b,c.

### 3.4. Plastic Anisotropy in Tension

It is postulated that ND fiber orientations improve deep drawing properties in steel sheets [27]. Two different values are used to assess deep drawing properties: average normal anisotropy coefficient (*r*_m_), which determines the limiting draw ratio, and planar coefficient of anisotropy (Δr), which is associated with earing behavior [28]. A maximum *r*_m_ and a minimal $\left|\Delta r\right|$ are best for deep drawing application [26].

The values of *r*_m_ and Δ*r* can be defined as follows:

(1) $$r_{m}=\left(r_{0}+2r_{45}+r_{90}\right)/4$$

(2) $$\Delta r=\left(r_{0}-2r_{45}+r_{90}\right)/2$$

where *r*_0_, *r*_45_, and *r*_90_ are the tension ratio in the directions that formed angles of 0°, 45°, and 90°, respectively, with the RD. Table 3 shows that the *r*_m_ value of route B was larger than that of route A. Moreover, the $\left|\Delta r\right|$ of route B was smaller than that of route A. Based on grain morphology, the average grain size is considered to have a very small effect on the mechanical properties, assuming that the apparent diameter of grains from two dimensional sections will most likely be similar to their respective diameter in the case of three dimensions [29]. This optimum improvement in the deep drawability of route B compared with that of route A was most likely associated with the microstructure exhibiting homogeneous γ-fiber textures, which was achieved through the selected growth phenomenon of the nuclei formed through Σ7 boundaries.

### 3.5. Surface Ridging

During tension, ferritic STS generally exhibits severe ridging (the surface shows undulations) parallel to RD. Viana et al. suggested that the severe ridging can be associated with microstructure and texture inhomogeneity that causes grain buckling under stress [30]. The <111>//ND texture is well-known to be favorable for the forming properties and increasing the resistance toward ridging. On the other hand, the α-fiber orientations are generally believed to have a deleterious effect on formability and ridging [31,32,33], which call for a serious effort to be eliminated during the process.

In this study, the profiling of the surface of the samples after 15% tension along RD was carried out to characterize the surface ridging of sample routes A and B (Table 4). The surface profile showed that the arithmetical mean (R_a_) of route B (~0.80) was relatively smaller and uniform compared to that of route A (~1.07). From the calculation, the roughness (R_t_) value was smaller in the case of route B (5.64 ± 0.01) as compared to route A (8.37 ± 0.01). It is well known that recrystallization nuclei with random orientations played an important role, since they improve isotropic properties that in turn increase the plastic strain ratio [34,35]. However, the nuclei from the selective growth-controlled recrystallization were expected to effectively maximize *r_m_* while minimizing Δ*r* by generating a microstructure with homogeneous γ-fiber textures, which effectively increase the plastic strain ratio and most likely also improve the resistance to surface ridging.

## 4. Conclusions

In this study, the role of reduction sequence during rolling on deformed texture and anisotropy of ferritic stainless steel are investigated. At fixed final rolling reduction between route A (67% + 50%, total 83%) and route B (50% + 67%, total 83%), the difference in terms of average grain size was not significant. Based on EBSD analysis, the texture distributions of routes A and B showed almost similar profiles, where most orientations were distributed along the *γ*-fiber. However, route B leads to a microstructure with homogeneous and sharp textures on all components along the γ-fiber, while in route A, the texture was less homogeneous, and the intensity of (111)[0–11] seemed to be missing. The homogeneous textures in route B improved both the average *r*-value and the planar anisotropy by maximizing the value of *r*_m_ (to a value near 2) while minimizing the value of Δ*r* (~0.87), respectively. Moreover, the sample processed via route B exhibited a better resistance toward ridging based on the smaller values of both R_a_ and R_t_ as compared to that processed via route A. The resulting microstructure with a homogeneous distribution of <111>//ND components was explained in relation to the formation of CSL boundaries of Σ7, which triggered the occurrence of selective growth-controlled recrystallization.

## Figures and Tables

**Table 1:** Chemical composition of the present ferritic STS (mass%).

C	N	Si	Mn	Ni	Cr	Ti	Fe
0.006	0.008	0.156	0.276	0.115	17.463	0.273	Bal.

**Table 2:** Processing history of different routes.

Route	Initial Thickness	1st Rolling	IA	2nd Rolling	FA	Final Thickness	Total Reduction
A	3.0 mm	67% (5-pass)	950 °C, 45 s	50% (3-pass)	950 °C, 45 s	0.5 mm	83%
B	3.0 mm	50% (3-pass)	950 °C, 45 s	67% (5-pass)	950 °C, 45 s	0.5 mm	83%

**Table 3:** *r*-values in the 0°, 45°, and 90° directions ($r_{0},\;r_{45},\;r_{90}$) from RD, *r_m_*, and Δ*r* with route A and route B of ferritic STS.

Route		*r*-Value		*r_m_*	Δ*r*
A	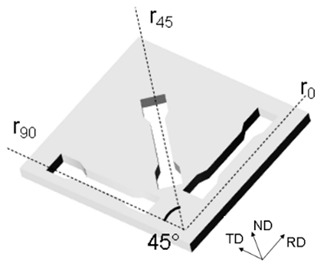	*r*_0_	1.49 ± 0.01	1.69 ± 0.003	0.89 ± 0.05
		*r*_45_	1.24 ± 0.01		
		*r*_90_	2.78 ± 0.01		
B		*r*_0_	2.25 ± 0.01	1.93 ± 0.02	0.87 ± 0.045
		*r*_45_	1.50 ± 0.01		
		*r*_90_	2.50 ± 0.01		

**Table 4:** Ridging topography under uniaxial tension in the RD at 15% elongation.

Route	Ridging Topography	Roughness (R_t_ = y_max_ − y_min_) (μm)
A	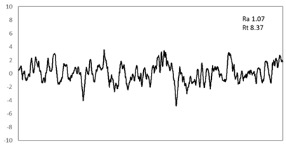	8.37 ± 0.01
B	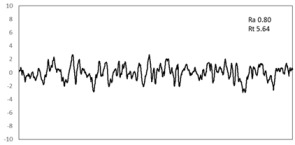	5.64 ± 0.01

## Data Availability

The data presented in this study are contained within the article.
